# Pulsed Thulium:YAG laser – What is the lithotripsy ablation efficiency for stone dust from human urinary stones? Results from an in vitro PEARLS study

**DOI:** 10.1007/s00345-023-04640-4

**Published:** 2023-10-13

**Authors:** Jia-Lun Kwok, Eugenio Ventimiglia, Vincent De Coninck, Frédéric Panthier, Yazeed Barghouthy, Alexandre Danilovic, Anil Shrestha, Niamh Smyth, Florian Alexander Schmid, Manuela Hunziker, Cédric Poyet, Michel Daudon, Olivier Traxer, Daniel Eberli, Etienne Xavier Keller

**Affiliations:** 1 Department of Urology, University Hospital Zurich, University of Zurich, Frauenklinikstrasse 10, 8091 Zurich, Switzerland; 2 Department of Urology, Tan Tock Seng Hospital, Singapore, Singapore; 3 Progressive Endourological Association for Research and Leading Solutions (PEARLS), Paris, France; 4 Endourology & Urolithiasis Working Group, Young Academic Urologists (YAU), Arnhem, The Netherlands; 5 Division of Experimental Oncology/Unit of Urology, Urological Research Institute, IRCCS Ospedale San Raffaele, Milan, Italy; 6 Department of Urology, AZ Klina, Brasschaat, Belgium; 7 GRC N°20, Groupe de Recherche Clinique Sur La Lithiase UrinaireHôpital Tenon, Sorbonne Université, 75020 Paris, France; 8 Department of Urology, Centre Hospitalier de Valenciennes, Valenciennes, France; 9 Department of Urology, Universidade de São Paulo Hospital das Clínicas—HCUSP, São Paulo, Brazil; 10 Department of Urology, Hospital Alemão Oswaldo Cruz, São Paulo, Brazil; 11 Department of Urology, National Academy of Medical Sciences, Bir Hospital and B&B Hospital, Gwarko Lalitpur, Nepal; 12 University Hospital Monklands, Monkscourt Avenue, Airdrie, ML60JS UK; 13 Hôpital Tenon, CRISTAL Laboratory, Sorbonne Université, Paris, France

**Keywords:** Laser, Pulsed Thulium:YAG, Kidney stones, Ablation, Endourology, Ureteroscopy, Dust

## Abstract

**Background:**

The novel pulsed thulium:yttrium–aluminum–garnet (p-Tm:YAG) laser was recently introduced. Current studies present promising p-Tm:YAG ablation efficiency, although all are based on non-human stone models or with unknown stone composition. The present study aimed to evaluate p-Tm:YAG ablation efficiency for stone dust from human urinary stones of known compositions.

**Methods:**

Calcium oxalate monohydrate (COM) and uric acid (UA) stones were subjected to lithotripsy in vitro using a p-Tm:YAG laser generator (Thulio®, Dornier MedTech GmbH, Germany). 200 J was applied at 0.1 J × 100 Hz, 0.4 J × 25 Hz or 2.0 J × 5 Hz (average 10W). Ablated stone dust mass was calculated from weight difference between pre-lithotripsy stone and post-lithotripsy fragments > 250 µm. Estimated ablated volume was calculated using prior known stone densities (COM: 2.04 mg/mm^3^, UA: 1.55 mg/mm^3^).

**Results:**

Mean ablation mass efficiency was 0.04, 0.06, 0.07 mg/J (COM) and 0.04, 0.05, 0.06 mg/J (UA) for each laser setting, respectively. This translated to 0.021, 0.029, 0.034 mm^3^/J (COM) and 0.026, 0.030, 0.039 mm^3^/J (UA). Mean energy consumption was 26, 18, 17 J/mg (COM) and 32, 23, 17 J/mg (UA). This translated to 53, 37, 34 J/mm^3^ (COM) and 50, 36, 26 J/mm^3^ (UA). There were no statistically significant differences for laser settings or stone types (all *p* > 0.05).

**Conclusion:**

To our knowledge, this is the first study showing ablation efficiency of the p-Tm:YAG laser for stone dust from human urinary stones of known compositions. The p-Tm:YAG seems to ablate COM and UA equally well, with no statistically significant differences between differing laser settings.

## Introduction

The prevalence of urinary stone disease has been increasing over the past decade, and in tandem so has the volume of endourological surgeries to treat the disease burden [1]. Within the field of endourological surgery, the laser has been established as the main tool for lithotripsy over the last three decades [2]. For laser surgical techniques, stone dusting has become increasingly popular together with newest generation lasers [3–5].

Currently, the holmium:yttrium–aluminum–garnet (Ho:YAG) and the more recent thulium fiber laser (TFL) are widely used in endourological procedures [6–8], with their effectiveness against human urinary stones extensively evaluated [9–14].

Recently, novel pulsed thulium:yttrium–aluminum–garnet (p-Tm:YAG) lasers have been introduced to the market for clinical use. Studies on p-Tm:YAG ablation found in literature so far have shown promising data, although all these studies were based on artificial stone lithotripsy models (BegoStone, plaster of Paris, gypsum/glass) [15–17]. It was only until recently that the p-Tm:YAG was shown capable to ablate the most common human urinary stone types [18]. Two recent studies evaluated the clinical efficacy and safety of the p-Tm:YAG on case series of patients undergoing retrograde intrarenal surgery [19] and mini-percutaneous nephrolithotomy [20]. Both studies conclude that the p-Tm:YAG seems very promising, although no information relating to its applicability to differing stone compositions was provided.

With the above background, the aim of the present study was to evaluate the currently unexplored question of p-Tm:YAG ablation efficiency for stone dust from human urinary stones of two known compositions.

## Materials and methods

Human urinary stones of two compositions were obtained from a stone biobank at Tenon Hospital, Paris: calcium oxalate monohydrate (COM) and uric acid (UA). Stones were chosen to match a diameter of about 5 mm, accepting that not all stones were perfectly spherical. This was in a comparable size range with other in vitro Ho:YAG and TFL ablation studies using human kidney stones [21–24] and stone phantoms [25, 26]. To simulate in vivo settings, all stones were immersed in saline for 24 h prior to experiments, due to kidney stones being of a crystalline structure primarily, but growing in a biological environment with complex intercrystalline spaces likely filled with urine [27].

Each stone separately underwent laser lithotripsy using the Dornier Thulio^®^ p-Tm:YAG with its 270 µm core-diameter Dornier Thulio^®^ Performance reusable laser fiber (Dornier MedTech GmbH, Wessling, Germany).

Since optimal settings for the production of stone dust using the p-Tm:YAG were not known at the time of this study, we explored several different laser settings. For each sample submitted to lithotripsy, a cumulative energy of 200 J was applied, with the use of one of three laser settings: 0.1 J × 100 Hz, 0.4 J × 25 Hz, and 2.0 J × 5 Hz. All chosen settings resulted in an average power of 10W. For each stone sample, as the average power used (10W) was the same to reach a common cumulative energy (200J), the lasing time was the same throughout all experiments. As suggested by the graphical user interface (GUI), the “Dusting” mode was used for the lower pulse range (the GUI proposes a pulse energy ranging from 0.1 J to 0.5 J in this mode), whereas the “Standard Fragmenting” mode was used for the higher pulse range (the GUI proposes a pulse energy ranging from 0.6 J to 2.0 J in this mode). For the 0.1 J × 100 Hz setting, we chose the lowest pulse energy possible (0.1 J) on the GUI in “Dusting” mode with a corresponding frequency (100 Hz) to reach a power of 10W. For the 0.4 J × 25 Hz setting, the pulse energy was matched to a generally accepted dusting setting (0.4 J × 25 Hz) for lithotripsy with Ho:YAG and TFL (GUI “Dusting” mode) [4, 28]. The third 2.0 J × 5 Hz setting was chosen to evaluate high pulse energy lithotripsy, meeting the previously set 10W average power (GUI “Standard Fragmenting” mode). As pulse duration was not displayed on the GUI and could not be changed within the operating modes themselves, this laser setting was not further explored. For each of the three laser settings and for each two stone types, lithotripsy was repeated separately with 5 urinary stones resulting in 5 measurements per laser setting and stone type (total of 30 samples).

Laser lithotripsy was performed under direct endoscopic vision in a 10 ml glass cuvette using the OTU WiScope (OTU Medical Inc, CA, USA) flexible ureteroscope, with sterile 0.9% sodium chloride saline irrigation at room temperature (21 °C) and constant irrigation pressure (40 cmH_2_O). Before lithotripsy of each stone sample, the laser fiber tip was cut with regular metal surgical scissors through the protective blue jacket. We used the same reusable fiber for all experiments performed. During lithotripsy, the laser fiber tip was continuously maintained as close as possible to the surface of the stones (i.e., working distance as short as possible).

Primary outcome was ablation mass efficiency defined as ablated stone dust mass per unit of laser energy (mg/J). Ablated stone dust mass was calculated from the difference in weight between the pre-lithotripsy stone and post-lithotripsy remnant fragments, in reminiscence of prior studies using this method to evaluate ablation parameters [17, 29–32]. Post-lithotripsy fragments were defined as particles > 250 µm, considering a prior study defining stone dust as stone particle ≤ 250 µm [33]. For that purpose, each post-lithotripsy sample was separately passed through a 250 µm mesh size laboratory sieve (Eisco sorting sieve, Eisco Scientific LLC, NY, USA), and poured with a total of 500 ml saline to isolate remnants fragments from filtered-off stone dust. For weighing of stones and remnant fragments, samples were dried with dabbing of filter paper to remove excess saline, then weighed with a laboratory balance AX105DR Analytical Balance (Mettler-Toledo GmbH, Greifensee, Switzerland).

Secondary outcome was estimated ablation volume efficiency in terms of ablated volume per unit of energy (mm^3^/J), to relate with prior studies that evaluated ablated volume rather than weight in vitro [9, 34, 35]. For calculation of estimated ablated stone volume, stone density values of 2.038 mg/mm^3^ for COM and 1.546 mg/mm^3^ for UA were used, respectively, based on measurements from a prior study using a pycnometer for evaluation of human urinary stone density [36]. The estimated ablated volume was then calculated by ablated stone dust mass divided by stone density (volume = mass / density).

Finally, ablation mass efficiency (mg/J) and ablation volume efficiency (mm^3^/J) were converted to energy consumption (J/mg and J/mm^3^, respectively). This allowed evaluation of the secondary outcome from the perspective of “How much energy do we need to ablate 1 mm^3^ of stone”, to relate with prior studies that evaluated laser energy consumption rather than laser efficiency [9, 10, 14, 37–44].

### Statistical analysis

Analyses comparing the three laser settings for each stone composition type were performed using one-way ANOVA with Tukey post hoc comparisons. Unpaired *t* test analyses were performed to evaluate pre-lithotripsy weight, ablation efficiency, and energy consumption between COM and UA stones. A two-sided *p* value < 0.05 was considered statistically significant. All descriptive and statistical analyses were performed with GraphPad Prism 9.5.1 (GraphPad Software, La Jolla CA, USA).

## Results

### Ablated stone dust mass

Mean stone weight before lithotripsy for COM was 22 mg (95% CI 20–25) vs. UA 27 mg (95% CI 23–31) (*p* = 0.07). Mean ablated stone dust mass with 200 J of laser energy was 8.4, 11.8, 13.9 mg for COM (ANOVA *p* = 0.08) and 8.1, 9.3, 12.1 mg for UA (ANOVA *p* = 0.11) for the laser settings 0.1 J × 100 Hz, 0.4 J × 25 Hz, 2.0 J × 5 Hz, respectively (Table 1).

**Table 1:** p-Tm:YAG ablation efficiency for stone dust from human urinary stones

	Stone composition	Laser setting (200 J@10W)			One-way ANOVA for laser settings within each stone composition
		0.1 J × 100 Hz	0.4 J × 25 Hz	2.0 J × 5 Hz	
Ablated stone dust mass (mg) (95% CI)	COM	8.4 (5.1–11.7)	11.8 (7.9–15.8)	13.9 (8.2–19.6)	*p* = 0.08
	UA	8.1 (3.9–12.3)	9.3 (5.7–12.9)	12.1 (9.5–14.8)	*p* = 0.11
	Student’s *t* test for each laser setting	*p* = 0.89	*p* = 0.22	*p* = 0.45	–
Ablation mass efficiency (mg/J) (95% CI)	COM	0.042 (0.025–0.058)	0.059 (0.039–0.079)	0.070 (0.041–0.098)	*p* = 0.08
	UA	0.041 (0.019–0.062)	0.046 (0.028–0.064)	0.061 (0.047–0.074)	*p* = 0.11
	Student’s *t* test for each laser setting	*p* = 0.89	*p* = 0.22	*p* = 0.45	–
Ablation volume efficiency* (mm^3^/J) (95% CI)	COM	0.021 (0.012–0.029)	0.029 (0.019–0.039)	0.034 (0.020–0.048)	*p* = 0.08
	UA	0.026 (0.013–0.040)	0.030 (0.018–0.042)	0.039 (0.031–0.048)	*p* = 0.11
	Student’s *t* test for each laser setting	*p* = 0.35	*p* = 0.86	*p* = 0.42	-

^*^Volume estimated using prior reported stone density of natural human urinary stones[36]

### Ablation efficiency

Overall ablation efficiency was 0.053 mg/J (95% CI 0.046–0.060), translating to 0.030 mm^3^/J (95% CI 0.026–0.034). When considering only COM samples, overall ablation efficiency was 0.057 mg/J (95% CI 0.046–0.068), translating to 0.028 mm^3^/J (95% CI 0.022–0.033). For UA samples, overall ablation efficiency was 0.049 mg/J (95% CI 0.040–0.058), translating to 0.032 mm^3^/J (95% CI 0.026–0.037).

**Fig. 1:**
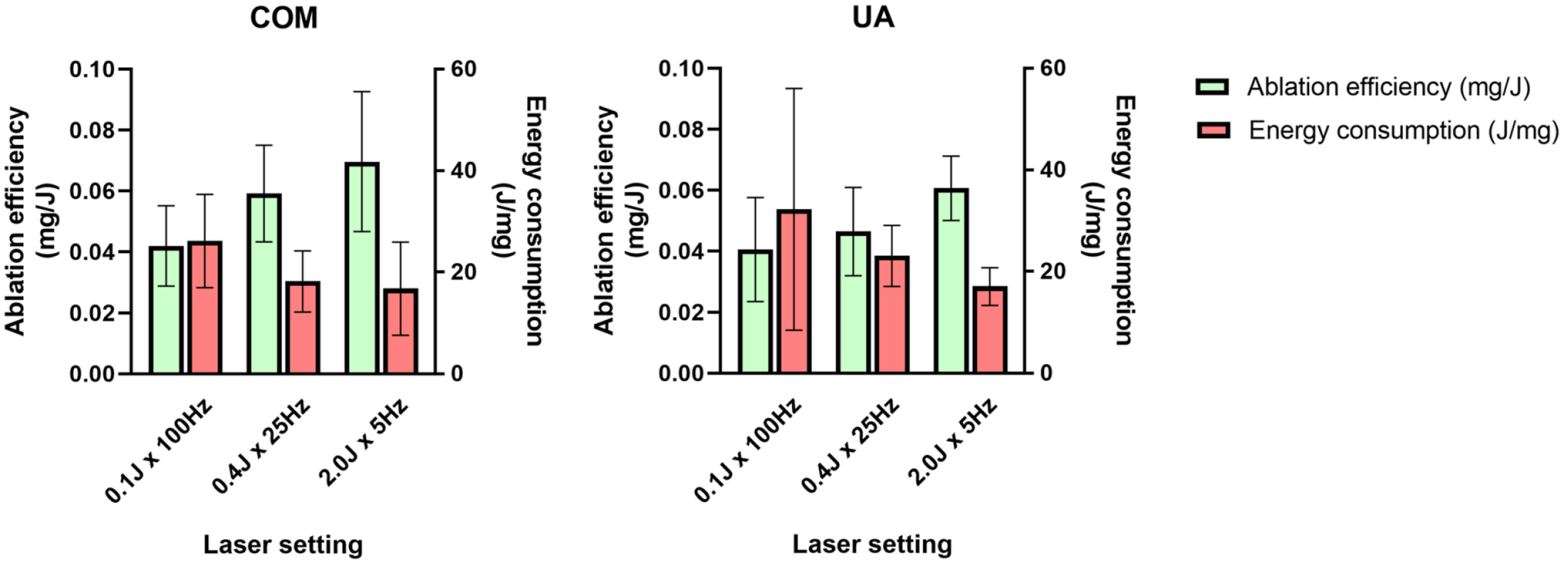
p-Tm:YAG laser ablation mass efficiency (mg/J) and energy consumption (J/mg) for stone dust

**Fig. 2:**
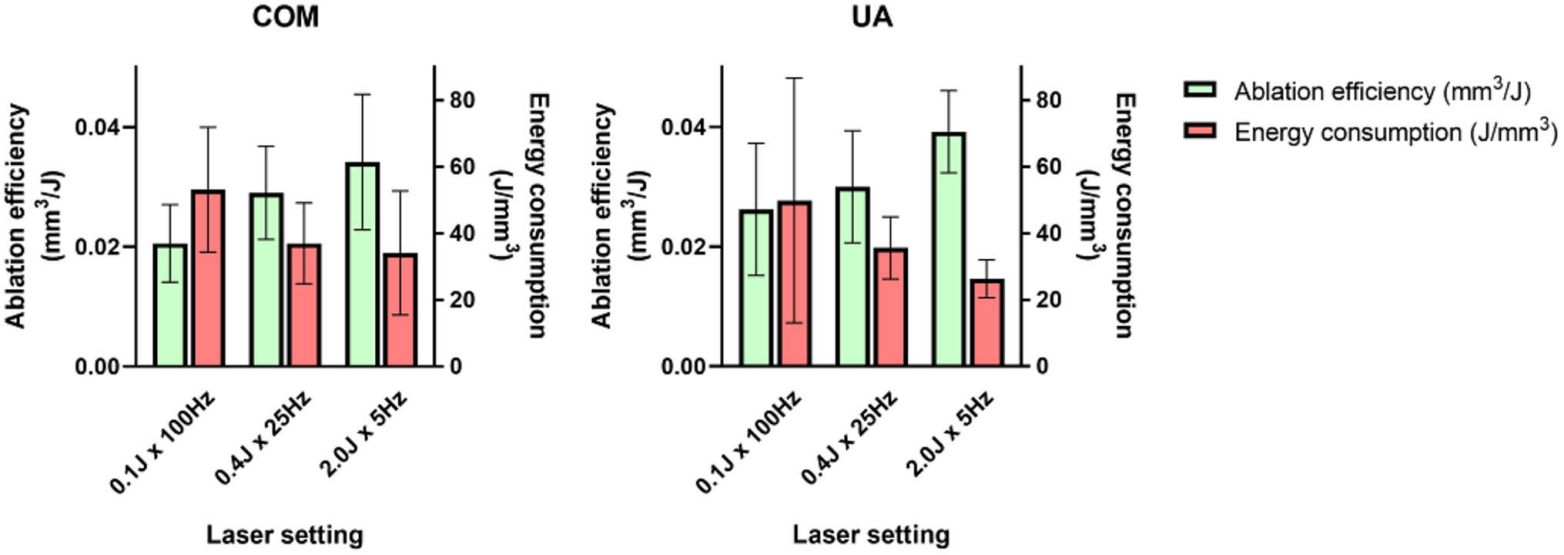
p-Tm:YAG laser ablation volume efficiency (mm^3^/J) and energy consumption (J/mm^3^) based on estimated ablated stone dust volume

Comparisons of ablation efficiency for each laser setting and stone type are summarized in Table 1, as well as in Fig. 1 (mass) and Fig. 2. (volume). There were no significant differences of ablation efficiency between settings for both COM and UA (ANOVA *p* = 0.08 and *p* = 0.11 respectively). Likewise, when comparing stone compositions within each laser setting, there were no significant differences (all *p* > 0.05).

### Energy consumption

Overall energy consumption was 22 J/mg (95% CI 18–27), translating to 39 J/mm^3^ (95% CI 32–47). For COM, this was 20 J/mg (95% CI 15–25), translating to 41 J/mm^3^ (95% CI 32–51), and for UA 24 J/mg (95% CI 16–32), translating to 37 J/mm^3^ (95% CI 25–50). Comparisons of estimated energy consumption for each laser setting and stone type are summarized in Table 2, Fig. 1 (mass), and Fig. 2 (volume). In analogy to laser ablation efficiency comparisons, there were no significant differences of energy consumption between COM and UA for laser settings (ANOVA *p* = 0.20 and *p* = 0.27, respectively). Likewise, when comparing stone compositions within each laser setting, there were no significant differences (all *p* > 0.05).

**Table 2:** p-Tm:YAG energy consumption for stone dust from human urinary stones

Mean measurement	Stone composition	Laser setting (200 J@10W)			One-way ANOVA for laser settings within each stone composition
		0.1 J × 100 Hz	0.4 J × 25 Hz	2.0 J × 5 Hz	
Energy per ablated mass (J/mg) (95% CI)	COM	26.1 (14.7–37.5)	18.2 (10.8–25.6)	16.8 (5.4–28.1)	*p* = 0.20
	UA	32.2 (2.7–61.8)	23.0 (15.5–30.5)	17.0 (12.4–21.6)	*p* = 0.27
	Student’s *t* test for each laser setting	*p* = 0.61	*p* = 0.24	*p* = 0.95	–
Energy per estimated ablated volume* (J/mm^3^) (95% CI)	COM	53.2 (29.9–76.5)	37.0 (21.9–52.2)	34.1 (11.0–57.3)	*p* = 0.20
	UA	49.8 (4.2–95.5)	35.6 (24.0–47.2)	26.3 (19.2–33.4)	*p* = 0.27
	Student’s *t* test for each laser setting	*p* = 0.86	*p* = 0.84	*p* = 0.40	–

^*^Volume estimated using prior reported stone density of natural human urinary stones[36]

## Discussion

To the best of our knowledge, this is the first study evaluating ablation efficiency of the p-Tm:YAG laser for stone dust from human urinary stones of different compositions. The p-Tm:YAG laser appears to ablate COM and UA stones equally well, with no significant differences between differing laser settings.

Additionally, this study provides a unique methodology accounting for the quality of ablated stone mass (i.e., particles ≤ 250 µm). Prior studies that used volumetric measurements in highly standardized in vitro settings (e.g., single-pulse craters, or fissures on perfectly flat artificial stones) [9, 17, 34, 35] completely omitted the quality assessment of pulverized stone material. Larger stone fragments may arguably have chipped-off the “main stone” during lithotripsy and falsely account for ablated stone volume [32]. Super-standardized study setups, with fixed standoff distance of the laser fiber tip not adjusted throughout the lithotripsy process, may not entirely reflect real-life laser application. Therefore, we deliberately chose a study setting simulating in vivo lithotripsy with free-hand application, where the targeting of stones’ surfaces will never be as standardized as it can be set in vitro.

Regarding choice of ablation efficiency measurement units, there is no current consensus on standardized terminology. Therefore, we chose mg/J and mm^3^/J units to reflect that a higher value would represent a more efficient process of laser lithotripsy. This is opposed to using energy consumption (J/mg and J/mm^3^) [34], where counterintuitively the higher the value, the worse the ablation efficiency. For the sake of readability and considering that several prior studies reporting their results as J/mm^3^ rather than mm^3^/J [9, 14, 37, 38], we provided our results considering both energy consumption and laser efficiency measurement units. The energy consumption was in the range 26.3–53.2 J/mm^3^ in the present study, translating to a laser efficiency of 0.021–0.039 mm^3^/J. These measurements compare with a wide range of different energy consumption outcomes reported for Ho:YAG and TFL lasers, ranging from 2.0–43.5 J/mm^3^ in vitro and 2.7–47.8 J/mm^3^ in vivo [45]. Direct comparison of results between these studies seems hazardous, unless standardized lasering conditions and outcome measurement methods are applied (including stone composition types, volume, density, laser settings, and total energy). Thus, further in vivo studies are warranted to compare ablation efficiency between these three lasers on human urinary stones head-to-head under the same conditions.

Worth mentioning, the methodology of our study required stone weight measurement as a primary outcome, while clinical decisions are mostly based on stone size metrics (ideally stone volume). Therefore, ablated stone mass was converted to estimated ablated stone volume. Considering ablated stone volume rather than mass, the general pattern of higher ablated stone weight favoring COM translated to a higher ablated stone volume favoring UA instead. Thus, evaluation of laser performance should always include analysis and discussion of several important metrics, including stone mass, density, and volume.

The optimal settings for ablation of human urinary stones with the p-Tm:YAG is currently unknown. It is interesting to note that there is a non-significant pattern for better ablation efficiency favoring a higher pulse energy (2.0 J × 5 Hz), followed by the next lower pulse energy (0.4 J × 25 Hz) and finally worst ablation efficiency for the lowest pulse energy setting (0.1 J × 100 Hz). Prior in vitro studies on evaluation versions of the p-Tm:YAG using BegoStone plates had similar findings of increased ablation efficiency when single-pulse energy was increased [16, 17]. Our study setup additionally accounts for some degree of retropulsion at higher pulse energies, and as aforementioned uses strict quality assessment of pulverized stone material for particle size meeting a known stone dust definition. This is not accounted for in the prior p-Tm:YAG study setups using fixed stone plates measuring effects of ablation craters [16, 17], or with omittance of chipped-off stone particle size assessment [17]. Furthermore, limitations of using BegoStone as compared to human stones are stated as limitations in one study [16]. No randomized clinical trial comparing laser settings and stone compositions for the p-Tm:YAG is currently available, and this should be further explored in vivo. When choosing laser settings, there are other considerations besides laser ablation efficiency. Based on clinical experience, the surgeon should be aware that a higher pulse energy may be associated with higher risk of stone retropulsion and mucosal bleeding. High frequency settings may impair visibility and cause poorer control on the target, which might be particularly harmful when lasering with high frequency in the ureter.

Of final note, COM is generally accepted as the “harder” stone to ablate compared to UA [9, 32, 46, 47]. Astonishingly no significant differences in ablation efficiency were found between these two stone types in the present study. This is analogous to a recent study where the high power Ho:YAG MOSES technology was found to ablate stones equally well, independent of stone density or composition type [14]. It is not known if the newest generation laser might ablate all stone types equally. This is a desirable property that needs to be further evaluated, with potential clinical implications affecting choice of laser and preoperative planning.

The study has several potential limitations. First, the present study is an in vitro attempt to assess p-Tm:YAG laser lithotripsy ablation efficiency that may impact in vivo use of the laser. The interpretation of data must be taken with care since environmental and surgical factors may impact clinical translation of the findings. Second, the sizes of the initial human urinary stones submitted to lithotripsy were rather small and not standardized, although this limitation is inherent to the use of human urinary stones, in reminiscence of prior in vitro Ho:YAG and TFL ablation studies using human kidney stones [21–24]. We balanced the necessity of repeated measurements using comparable human stones (5 repeated measurements × 3 laser settings × 2 stone types = total 30 samples) with the hypothetical ideal setup that was not possible to obtain (i.e., 30 stones samples of > 1 cm each). In addition, our study setup determined ablation performance measured by the stone weight difference pre-/post-lithotripsy. A recent randomized control trial comparing pulse-modulated Ho:YAG and TFL for renal and ureteral stones had small average stone sizes as well (largest stone diameter mean of 8.4/8.9 mm and median of 7.4/7.9 mm IQR [5.3–11.3]/[6.0–11.1] for each arm), reflecting that stones in real life, at least in the American setting and arguably the European setting, are not very large [48]. It would be interesting to repeat a similar study setup on several other stone compositions and if possible, larger stones in vivo to evaluate for differences. Third, rather than oven or freeze drying, the stones were dried with filter paper. However, the methodology was consistent for both pre- and post-lithotripsy samples, thus arguably mitigating the risks of a systematic bias. Finally, a limit of 200 J of energy was applied. As the mechanisms of relevance for lithotripsy with the p-Tm:YAG are not yet perfectly understood, further studies may be warranted looking at higher total energy applied. This may be closer to real world conditions.

## Conclusion

Based on our results, the p-Tm:YAG seems to ablate COM and UA stones equally well with no statistically significant differences, demonstrating good ablation regardless of composition or differing laser settings. Future perspectives to explore for the p-Tm:YAG include determining the best laser settings for dusting and fragmenting. Also, it will be important to compare the p-Tm:YAG with other laser technologies and further urinary stone types.

## Data Availability

On request to corresponding author for raw data on the experimental setup.
